# Photobiomodulation in Peripheral Nervous System Disorders: A Bibliometric Analysis of Functional Research Patterns

**DOI:** 10.3390/bioengineering13091006

**Published:** 2026-08-28

**Authors:** Ji-Woo Seok, Kahye Kim, Su-Jin Baek, Jin Mi Chun

**Affiliations:** 1 Digital Health Research Division, Korea Institute of Oriental Medicine, Yuseong-Daero 1672, Yuseong-Gu, Daejeon 34054, Republic of Korea; 2 Korean Medicine (KM) Data Division, Korea Institute of Oriental Medicine, Daejeon 34054, Republic of Korea

**Keywords:** photobiomodulation, peripheral nervous system, neuropathy, nerve regeneration, pain modulation, bibliometric analysis

## Abstract

This study provides a comprehensive bibliometric analysis of photobiomodulation (PBM) research in peripheral nervous system (PNS) disorders, aiming to characterize its structural features and developmental trends. Using a bibliometric workflow, a final dataset of 191 unique publications across 24 standardized PNS disease entities was identified through MeSH- and ICD-11-based classification from 14,670 records retrieved from the Web of Science Core Collection. The results show that PBM research has grown steadily since 2010 but remains concentrated in specific clinical conditions, particularly carpal tunnel syndrome (CTS). Keyword co-occurrence network analysis and functional classification identified three primary domains of PBM research: (1) pain modulation, (2) nerve regeneration and structural repair, and (3) biological/mechanistic regulation. The relative emphasis of these domains differed across disease groups, with compression and entrapment neuropathies emphasizing intervention parameters and assessment, nerve injury/regeneration research showing greater representation of structural repair and biological regulation, and neuropathic pain/neuropathy research showing a prominent pain-related component. Temporal analysis indicated diversification toward a broader range of neuropathic conditions and increasing representation of pain-related and biological themes. In conclusion, PBM research exhibits distinct functional patterns across PNS disease contexts. The function-oriented classification framework provides a structured basis for identifying research gaps and disease-specific research priorities.

## 1. Introduction

Peripheral nervous system (PNS) disorders are common neurological conditions caused by nerve damage, affecting approximately 1% of the global adult population [1,2]. The prevalence increases significantly to 6–10% among individuals aged 60 years and older [1,2]. Diabetic neuropathy represents one of the most prevalent forms of PNS disorders, affecting an estimated 206 million people worldwide and ranking as the fifth leading cause of disability burden among neurological diseases in 2021 [3].

The etiologies of PNS disorders are diverse, ranging from compressive mononeuropathies such as carpal tunnel syndrome to drug toxicity, infections, nutritional deficiencies, genetic factors, and trauma or burns [4]. These conditions typically manifest as numbness, sensory loss, and neuropathic pain, substantially impairing quality of life [4]. In advanced stages, they may lead to muscle atrophy and motor dysfunction, resulting in permanent disability [4].

Despite this substantial burden, current therapeutic approaches remain largely limited to symptomatic management, and complete functional recovery of damaged nerves remains challenging. For example, gabapentin, a commonly used pharmacological agent, achieves a 50% reduction in pain in only approximately 38% of patients, even at adequate doses [4]. These limitations underscore the need for non-invasive therapeutic strategies that can simultaneously promote nerve regeneration and alleviate pain [4].

Against this backdrop, photobiomodulation (PBM) has emerged as a promising therapeutic modality [5]. PBM utilizes low-level laser or light-emitting diode (LED) irradiation in the red to near-infrared spectrum to modulate cellular metabolism and tissue responses [5,6]. Because it employs non-ionizing light energy, PBM is considered safe, with a low risk of tissue damage. In studies of peripheral nerve injury, wavelengths between 630 and 905 nm are most commonly used [5,6], and clinical applications have reported minimal adverse effects [7].

Mechanistically, PBM influences neural function through multiple biological pathways, including mitochondrial activation and inflammatory modulation [8]. Recent studies have also suggested non-mitochondrial mechanisms, such as cytoskeletal dynamics and photophysical interactions [8]. These multimodal actions provide a biological basis for both neural regeneration and pain relief.

However, clinical outcomes of PBM remain inconsistent across studies. For example, a recent meta-analysis in patients with carpal tunnel syndrome demonstrated functional improvement but failed to show significant effects on pain or grip strength [9]. Such discrepancies are likely attributable to heterogeneity in treatment parameters, including wavelength, power, energy density, irradiation duration, and treatment frequency [9].

Collectively, these findings suggest that the therapeutic effects of PBM should be understood as a multi-layered functional process rather than a single mechanism [8]. Based on existing evidence, PBM effects in peripheral nerve injury can be categorized into three primary domains: (1) pain modulation [10], (2) neural repair and regeneration [11,12], and (3) biological and mechanistic regulation [13,14]. This functional categorization provides a conceptual basis for integrating the diverse therapeutic effects of PBM in PNS disorders.

Despite these insights, existing PBM research remains highly heterogeneous [15,16]. Study designs lack standardization, and key treatment parameters, such as wavelength, energy density, irradiation duration, and treatment frequency, vary substantially across studies [15,16]. In addition, mechanistic, preclinical, and clinical investigations have often progressed without integration and with inconsistent outcome measures, limiting comparability and hindering the development of optimized therapeutic strategies.

To address these gaps, this study aims to systematically reorganize PBM research through a functional perspective. Through bibliometric analysis, we aim to (1) classify PBM’s therapeutic effects by functional domain, (2) compare disease-specific application patterns, and (3) identify overall research trends and key knowledge gaps. Through this approach, we seek to consolidate fragmented evidence and establish a conceptual framework for characterizing disease-specific PBM research patterns and identifying research gaps in PNS disorders.

## 2. Methods

### 2.1. Study Design

This study aimed to characterize the research landscape of PBM for peripheral nervous system (PNS) disorders and to identify the structural characteristics of the existing literature. A bibliometric research design was employed using a systematically identified publication dataset retrieved from the Web of Science Core Collection (WoSCC). The analysis focused on quantifying publication characteristics, knowledge structure, disease distribution, and research trends related to PBM in PNS disorders. By integrating bibliometric mapping with disease-based classification, this study provides a comprehensive overview of the development and thematic organization of PBM research in this field.

### 2.2. Data Sources and Search Strategies

The bibliometric dataset was constructed using the Web of Science Core Collection (WoSCC), which served as the sole data source for all analyses. An intervention-focused search strategy was adopted to capture the full spectrum of PBM research without restricting specific disease categories. Search terms included “photobiomodulation”, “low-level laser therapy”, “LLLT”, “laser therapy”, and “phototherapy”. LED-related terminology (“light-emitting diode”, “light emitting diode”, “LED therapy”, and “LED irradiation”) was searched in combination with PBM-related terminology (“photobiomodulation”, “photobiomodulation therapy”, “low-level light therapy”, “low level light therapy”, or “phototherapy”). The search was performed in the Topic field of WoSCC. No restrictions were applied regarding publication year or document type, allowing original articles, reviews, and conference proceedings to be included. A total of 14,670 records were retrieved, and 14,552 unique publications remained after duplicate removal based on normalized article titles.

Disease-related information was extracted using a custom disease dictionary developed from the 2026 Medical Subject Headings (MeSH). Disease descriptors and entry terms corresponding to the disease category (tree numbers beginning with “C”) were used to construct the dictionary. Author Keywords and Keywords Plus were normalized and matched against the dictionary to identify disease names. Disease identification was based primarily on normalized exact matching to MeSH disease descriptors and clinically equivalent disease expressions, while PNS-specific MeSH entry terms were used to capture synonymous terminology within the target disease spectrum.

To improve disease specificity, overly broad symptom-level terms (e.g., “pain” and “inflammation”) were excluded. Semantically equivalent disease names were subsequently consolidated to establish standardized disease entities. Sciatica and lumbar, lumbosacral, and related radiculopathy expressions were standardized as radiculopathy. Similarly, synonymous expressions for peripheral neuropathies, nerve injuries, compression neuropathies, neuralgias, and related PNS conditions were mapped to standardized disease entities. When a broad PNS term and a more specific disease entity represented the same condition within a publication, the more specific disease label was retained to avoid hierarchical double counting.

**Figure 1:**
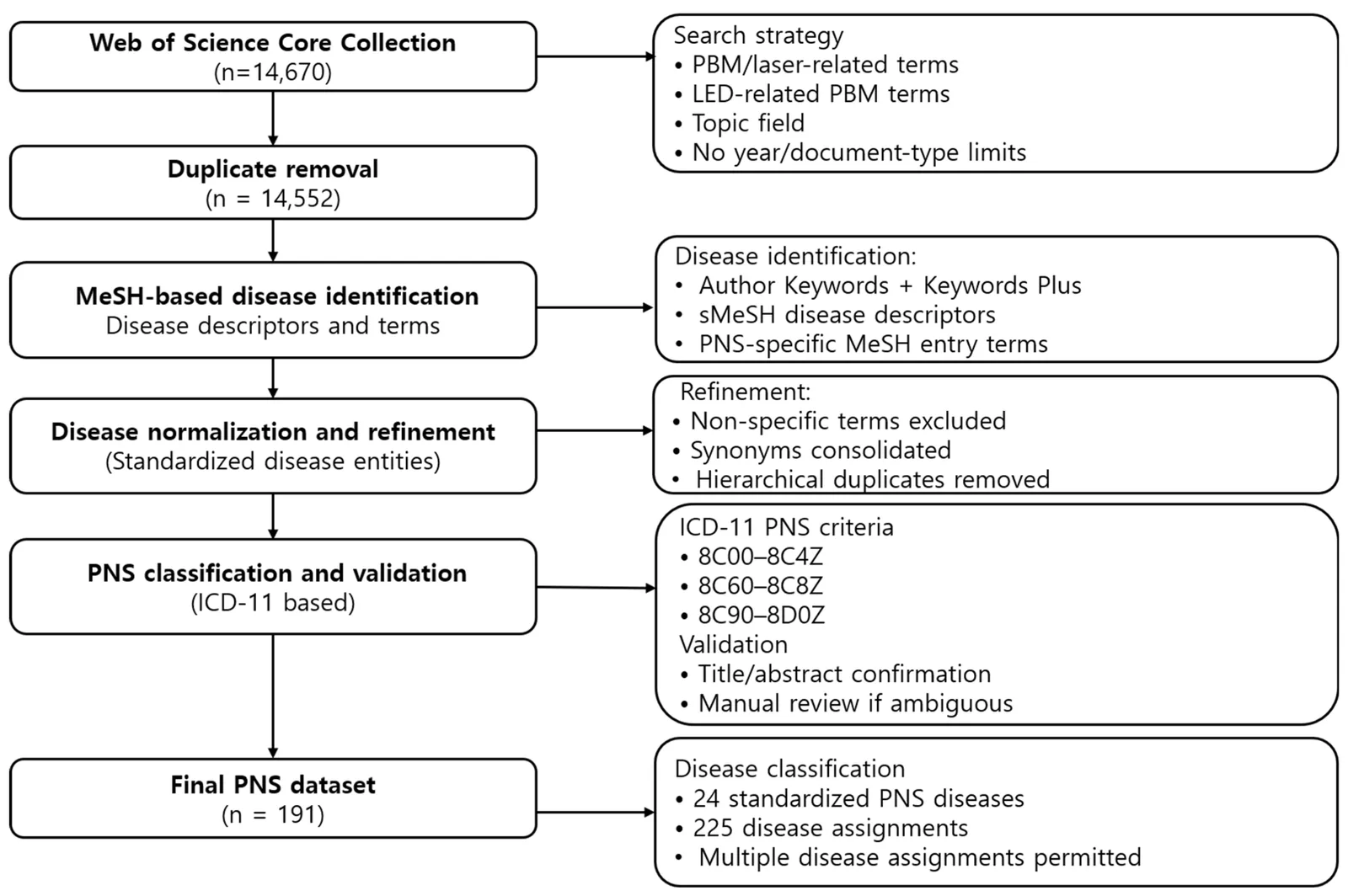
Flowchart of the literature selection and disease-specific data extraction process. Records retrieved from the Web of Science Core Collection were deduplicated, matched to MeSH disease terminology, normalized to standardized disease entities, and subsequently classified and validated according to ICD-11 PNS criteria. The final dataset comprised 191 unique publications across 24 standardized PNS disease entities, yielding 225 non-mutually exclusive disease assignments.

Publications related to PNS disorders were subsequently identified according to the International Classification of Diseases, 11th Revision (ICD-11). Disorders of nerve roots, plexuses, and peripheral nerves (8C00–8C4Z), polyneuropathies (8C60–8C8Z), and neuromuscular junction disorders (8C90–8D0Z) were included. Disease assignments were not mutually exclusive; a publication addressing more than one clinically distinct PNS disorder was therefore retained under each relevant disease entity. PNS disease assignments derived from Keywords Plus were verified against the corresponding title and abstract when the disease context was not otherwise evident, and ambiguous records were manually reviewed before final classification. Following synonym consolidation, hierarchical refinement, and PNS-specific validation, the final dataset comprised 191 unique publications across 24 standardized PNS disease entities. Because a publication could address more than one clinically distinct PNS disorder, the 191 publications yielded 225 disease assignments for disease-specific analyses. The complete workflow of dataset construction and disease classification is illustrated in Figure 1.

### 2.3. Data Extraction

Data were extracted from the final bibliometric dataset to identify bibliometric information and disease-specific research characteristics. For bibliometric analysis, we collected the title, authors, publication year, journal name, and author affiliations for each article. These data were used to analyze research productivity and the geographical distribution of research output.

To examine research themes and the knowledge structure, a consolidated dataset was constructed by integrating author keywords and Keywords Plus. Disease-related information for each article was organized using the matched disease variable, derived from MeSH-based matching and subsequent filtering according to ICD-11 criteria. Disease-specific analyses were performed at the publication–disease level; thus, a publication assigned to multiple clinically distinct PNS disorders contributed once to each applicable disease but was not counted more than once within the same disease.

### 2.4. Data Preprocessing

To enhance the accuracy of the bibliometric analysis, systematic data preprocessing and normalization were performed. All keywords and text data were converted to lowercase, and punctuation and redundant spaces were removed. Standardization was conducted to minimize semantic redundancy among terms representing the same concept. For example, synonymous PBM-related terms such as “low-level laser therapy,” “LLLT,” and “photobiomodulation” were consolidated into a single representative term. Equivalent diagnostic and assessment terminology was also standardized where appropriate; for example, ENoG/electroneurography and nerve conduction study terminology were harmonized, whereas electromyography was retained as a distinct assessment modality.

Additionally, non-informative or overly generic keywords (e.g., technical units such as nm, general intervention terms, and methodological descriptors) were removed to reduce noise in the dataset (Table S1). For keyword-frequency and co-occurrence analyses, the unit of occurrence was defined as a unique publication–keyword pair; therefore, repeated occurrences of the same normalized keyword within an individual publication were counted only once. For disease-specific analyses, the same principle was applied at the publication–disease–keyword level, such that a normalized keyword contributed at most once to a given disease within each publication.

### 2.5. Functional Classification of Keywords

To identify the structural characteristics and mechanisms of action in PBM research, extracted keywords were reclassified into a function-based framework tailored to the research context. Moving beyond simple bibliometric categorization, a function-based classification system was applied to reflect the therapeutic roles and biological effects of PBM. All keywords were assigned to one of the following seven predefined functional categories: pain modulation, structural repair, metabolic and biological regulation, diagnosis/assessment, clinical study design, technical/intervention parameters, and other.

The pain modulation category includes functions related to the modulation of neural excitability and pain transmission pathways, covering symptoms such as pain and paresthesia (e.g., neuropathic pain, allodynia, hyperalgesia). Keywords such as neuropathic pain, allodynia, hyperalgesia, paresthesia, and neuralgia were included to reflect symptomatic relief.

The structural repair category encompassed biological processes involved in the repair of damaged neural tissue and functional recovery, including nerve regeneration, remyelination, axonal repair, re-innervation, and Schwann cell activation.

The metabolic and biological regulation category captured PBM-related mechanisms at the cellular and molecular levels, including ATP production, mitochondrial activity, oxidative stress, inflammatory response, gene expression, and growth factors.

The diagnosis/assessment category included tools and indicators used to evaluate PBM effects, such as electromyography (EMG), nerve conduction studies (NCS), ultrasonography, clinical scales, and questionnaires.

The clinical study design category reflected methodological characteristics of studies, including randomized controlled trials (RCTs), double-blind designs, placebo-controlled studies, systematic reviews, and meta-analyses.

The technical/intervention parameters category comprised keywords describing the physical and technical properties of PBM, such as wavelength, energy density, laser type, and irradiation conditions.

Keywords that could not be clearly assigned to these categories or lacked functional specificity were classified as other. Disease-related keywords were handled separately. Primary disease terms used for classification were excluded to avoid redundancy, while secondary or comorbid conditions were retained but categorized as “other” without functional interpretation. Symptom-related terms were assigned to the pain modulation category based on their functional relevance.

Each keyword was assigned to a single category based on its primary functional role. Keywords were independently classified by two researchers according to the predefined functional classification criteria. Inter-rater agreement was 77.2%, with a Cohen’s κ of 0.70. Disagreements were resolved by consensus to determine the final category. For comparisons of functional research patterns across PNS disease groups, category proportions were calculated from classified keyword occurrences after excluding the “other” category from the denominator. The denominator therefore represented all classified functional keyword occurrences other than “other” within each disease group.

### 2.6. Bibliometric Analysis

Bibliometric analysis was performed using an R-based environment (e.g., bibliometrix) and VOSviewer (version 1.6.20) for visualization. Keyword frequency and co-occurrence analyses were conducted to evaluate the structural relationships among research topics. Disease-specific keyword frequencies were calculated as the number of unique publications containing each normalized keyword within each disease. A disease-by-keyword matrix was subsequently constructed from these publication counts and visualized as the primary heatmap. Each publication contributed only once to a given disease–keyword combination, regardless of repeated occurrences of the same normalized keyword. Thus, the heatmap represents the absolute publication frequency associated with each disease–keyword combination.

Network visualization and structural analysis were performed in VOSviewer, where node size represents keyword frequency, and the distance between nodes reflects the strength of association. For the construction of the keyword co-occurrence network, a thesaurus file was applied in VOSviewer to standardize synonymous terms and exclude non-informative or redundant keywords from the network visualization (Table S2). Author Keywords and Keywords Plus were analyzed together using full counting, and terms occurring in at least five publications were retained for network construction. After thesaurus-based normalization and filtering, 41 keywords were included in the final co-occurrence network. Link strength was normalized using the association strength method. Density visualization was used to identify areas of high research concentration, and overlay visualization was applied to examine temporal changes in research topics, with the color of each keyword representing its average publication year.

### 2.7. Analytical Framework

To compare the characteristics of PBM research across PNS disorders, diseases were categorized into three groups based on pathophysiological features: (1) compression and entrapment neuropathies, (2) nerve injury and regeneration, and (3) neuropathic pain and neuropathy.

Keyword distributions and proportions of functional categories were compared across groups to analyze differences in research focus according to disease type (e.g., clinical application, pain modulation, and regenerative mechanisms). Because individual publications could be assigned to more than one clinically distinct PNS disorder, disease-specific analyses retained these non-mutually exclusive assignments. Functional-category proportions were calculated from unique publication–disease–keyword occurrences within each disease group. This classification framework was used to identify structural differences in functional research patterns across disease groups.

## 3. Results

### 3.1. Overview of Included Publications

A total of 191 unique publications on PBM in PNS disorders were included in the final analysis, comprising 225 disease assignments across 24 standardized PNS disease entities. Publication years ranged from 2000 to 2026.

Original research articles represented the largest document type (*n* = 122), followed by reviews (*n* = 54), proceedings papers (*n* = 10), articles published as early access (*n* = 2), one article classified concurrently as a proceedings paper, one correction, and one meeting abstract. When document types were collapsed for visualization, the dataset comprised 126 articles, 54 reviews, and 11 proceedings publications.

At the disease-group level, the 225 disease assignments comprised 87 compression/entrapment assignments, 72 nerve injury/regeneration assignments, and 66 neuropathic pain/neuropathy assignments. Because disease assignments were non-mutually exclusive, these counts should not be interpreted as numbers of unique publications. At the unique-publication-within-group level, the corresponding numbers were 74, 61, and 61 publications, respectively.

### 3.2. Distribution of PBM Studies Across Diseases

Across 191 unique publications, PBM research is disproportionately concentrated in a limited number of diseases. Carpal tunnel syndrome was the most frequently represented condition, with 62 publications (32.5%).

Other commonly investigated conditions included facial paralysis (*n* = 27, 14.1%), peripheral nerve injuries (*n* = 27, 14.1%), diabetic neuropathy (*n* = 18, 9.4%), and trigeminal neuralgia (*n* = 13, 6.8%). Peripheral neuropathy (*n* = 10, 5.2%), radiculopathy (*n* = 8, 4.2%), and chemotherapy-induced peripheral neuropathy (*n* = 7, 3.7%) were also represented in the revised dataset.

Among additional conditions, polyneuropathy, reflex sympathetic dystrophy, trigeminal nerve injuries, and cranial nerve injuries each accounted for six publications, whereas cubital tunnel syndrome and nerve compression syndromes each accounted for five publications.

**Figure 2:**
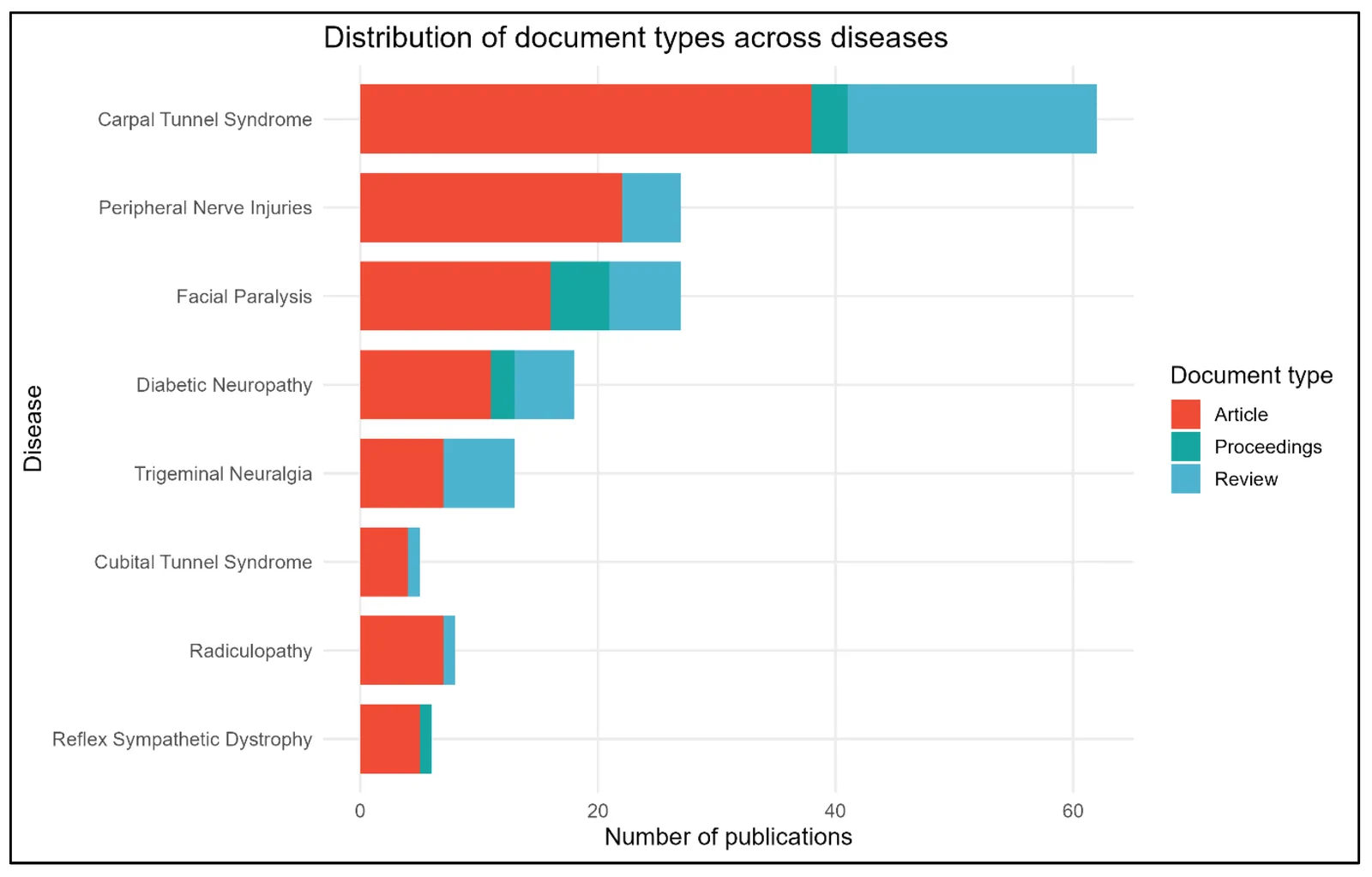
Distribution of PBM studies across major PNS diseases. Horizontal stacked bars show the number of unique publications for each major PNS disease according to document type. Disease assignments were not mutually exclusive; therefore, a publication addressing more than one clinically distinct PNS disorder could contribute to multiple disease categories.

Because publications could be assigned to more than one clinically distinct PNS disorder, disease-specific counts are non-mutually exclusive, and their sum exceeds the 191 unique publications. Overall, the distribution remained concentrated around carpal tunnel syndrome and a smaller set of frequently studied PNS disorders, while the revised classification captured a broader range of neuropathic and nerve-injury conditions (Figure 2).

### 3.3. Temporal Trends of PBM Research

**Figure 3:**
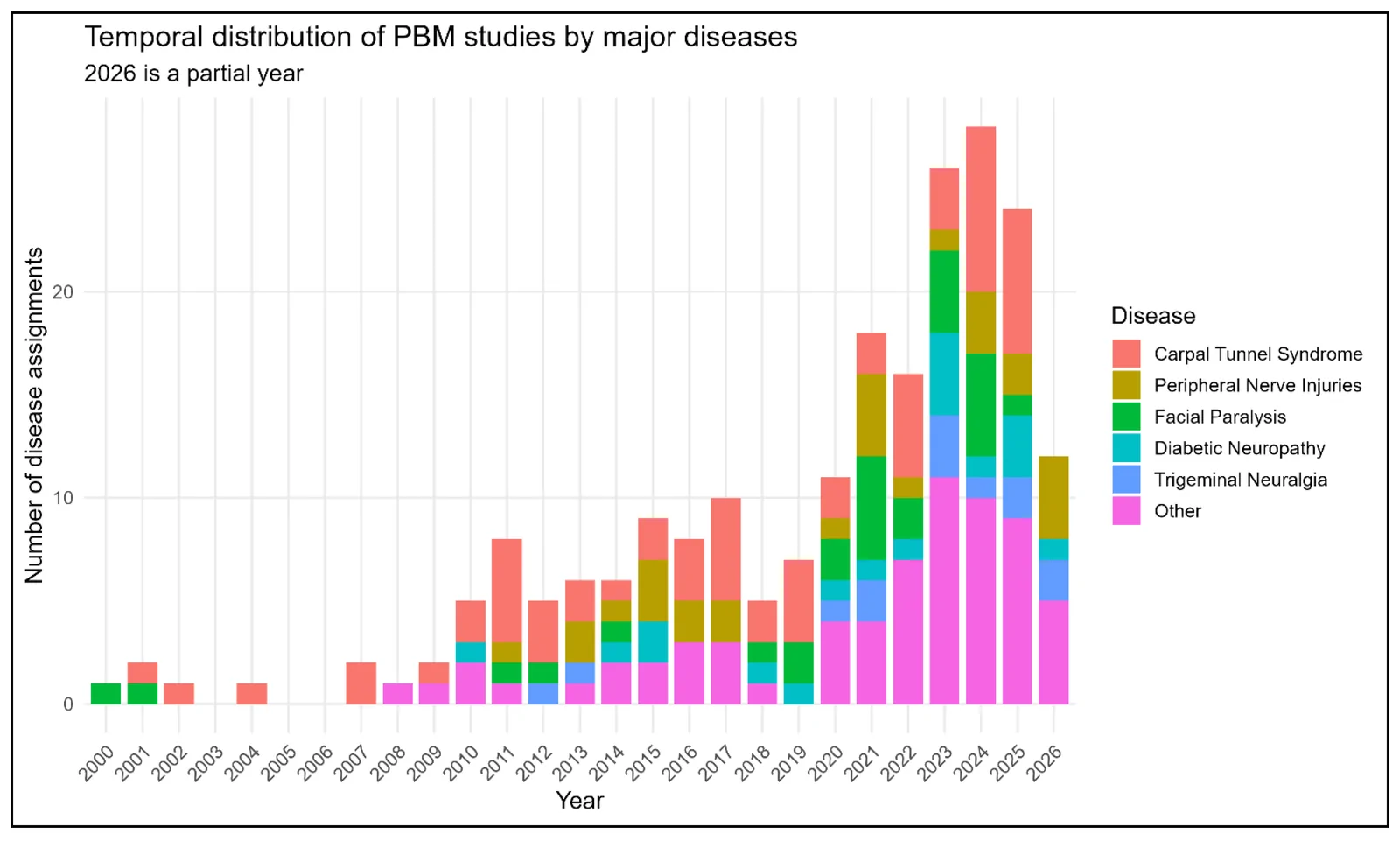
Temporal trends of PBM studies across major PNS disorders. Stacked bars represent annual disease assignments for the five most frequently studied PNS disorders, with all remaining conditions grouped as “Other.” Because individual publications could be assigned to more than one clinically distinct disease, the vertical axis represents disease assignments rather than unique publications. The 2026 data represent a partial year.

The annual publication trend indicates that PBM research on PNS disorders was limited in the early 2000s but has increased steadily since 2010. Specifically, 10 publications were published between 2000 and 2009, 54 between 2010 and 2017, and 127 between 2018 and 2026. The highest annual publication output was observed in 2024, with 25 publications, followed by 2023 with 22 publications (Figure 3). Because the 2026 search covered only part of the year, the 2026 count should not be interpreted as evidence of a decline in publication activity.

Disease-specific trends (Figure 3 and Figure S1) continued to show a prominent contribution from carpal tunnel syndrome throughout the study period. The expanded dataset also showed increasing representation of facial paralysis, peripheral nerve injuries, diabetic neuropathy, radiculopathy, and other neuropathic conditions during more recent years.

Importantly, the distribution of disease targets has broadened over time. Earlier PBM research was concentrated predominantly on carpal tunnel syndrome and peripheral nerve injury-related conditions, whereas the post-2018 literature encompassed a wider spectrum of compression neuropathies, neuropathic pain conditions, polyneuropathies, and nerve-injury disorders.

### 3.4. Journals and Geographic Distribution

Analysis of geographic research productivity demonstrated continued concentration in several countries. Brazil was the leading contributor with 49 publications, followed by China (*n* = 18), Iran (*n* = 17), Türkiye (*n* = 16), Taiwan (*n* = 11), and Egypt (*n* = 10) (Figure S2). Australia contributed nine publications and India eight, whereas Canada contributed seven. Brazil therefore remained the most prominent national contributor to PNS-related PBM research.

Disease- and disease-group-specific analyses further demonstrated geographic heterogeneity (Figures S3 and S4). Compression/entrapment research was most frequently represented in Türkiye (*n* = 13), followed by Brazil (*n* = 10) and China (*n* = 8). In contrast, nerve injury/regeneration research showed a marked concentration in Brazil (*n* = 28), while neuropathic pain/neuropathy research was also led by Brazil (*n* = 16), followed by India and Iran (*n* = 7 each). These findings indicate that geographic concentration differed according to the disease context being investigated.

The journal analysis revealed a pronounced concentration within specialized laser and photomedicine journals. *Lasers in Medical Science* was the most frequent publication venue (*n* = 30), followed by *Photobiomodulation, Photomedicine, and Laser Surgery* (*n* = 9); *Proceedings of SPIE* (*n* = 7); and *Photomedicine and Laser Surgery* (*n* = 6) (Figure S5).

Disease- and disease-group-specific journal distributions showed similar specialization (Figures S6 and S7). *Lasers in Medical Science* was the most frequently represented journal in all three disease groups, accounting for 12 compression/entrapment, 13 nerve injury/regeneration, and seven neuropathic pain/neuropathy publications. However, secondary publication venues differed across groups, reflecting variation in clinical, rehabilitation, mechanistic, and supportive-care research contexts.

At the institutional level, Universidade de São Paulo was the leading contributor (*n* = 20), followed by Cairo University and the Egyptian Knowledge Bank (*n* = 10 each). Harvard University, Harvard University Medical Affiliates, Tehran University of Medical Sciences, and Universidade Estadual Paulista each contributed six publications (Figure S8).

At the author level, productivity remained relatively distributed. Barraviera B, Chen YH, and Hamblin MR each contributed five publications, whereas Buchaim DV, Buchaim RL, Marcolino AM, Pinheiro ALB, and Wang Y each contributed four publications (Figure S9). Thus, the updated dataset continued to show a distributed author network rather than dominance by a single highly prolific investigator.

### 3.5. Keyword Network and Core Research Themes

**Figure 4:**
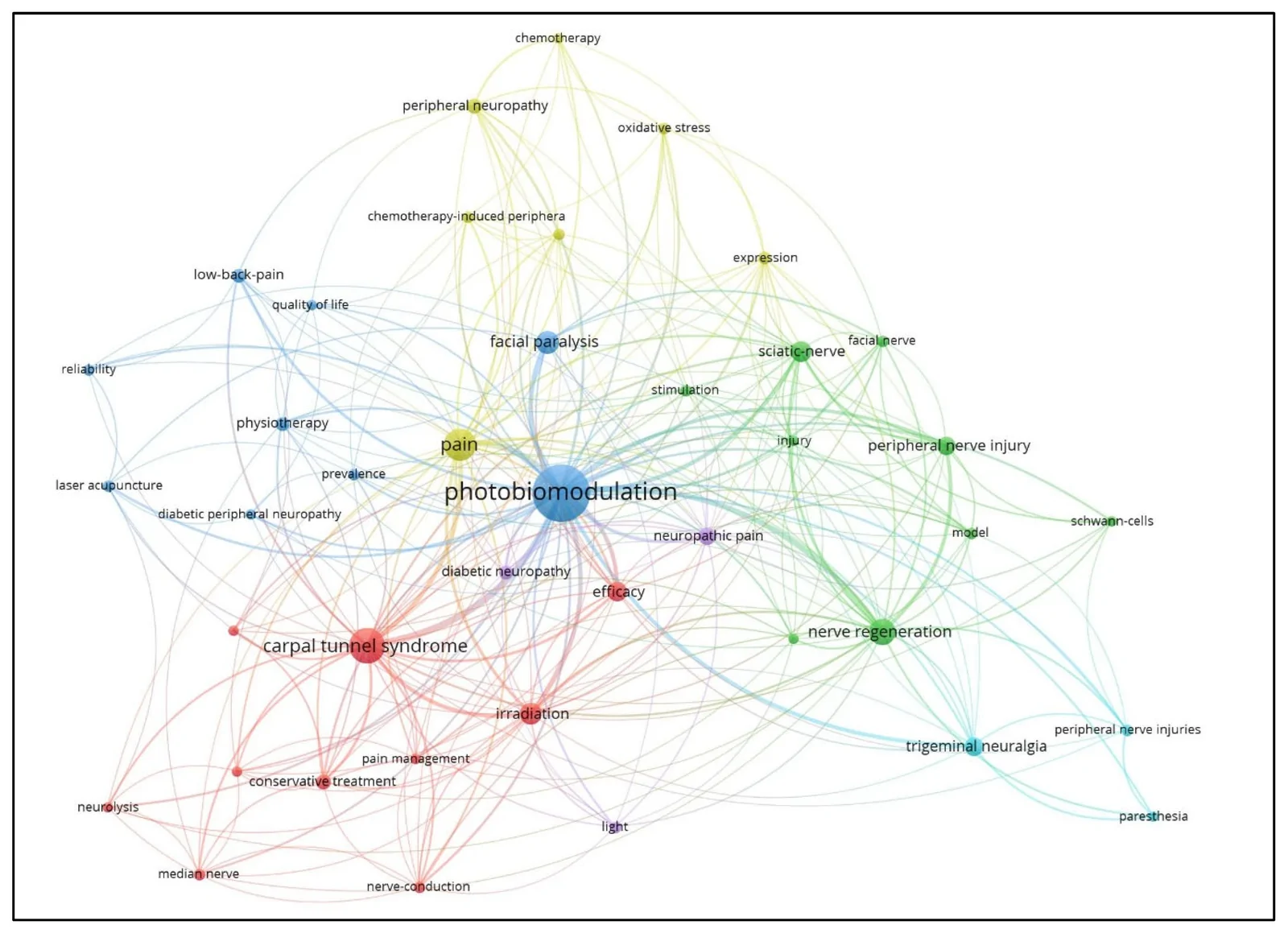
Keyword co-occurrence network of PBM research in PNS disorders. The network was constructed in VOSviewer using Author Keywords and Keywords Plus with full counting. Node size represents keyword occurrence frequency, links represent co-occurrence relationships, and colors indicate VOSviewer-derived keyword clusters. Synonymous and conceptually equivalent terms were standardized using a thesaurus, non-informative terms were excluded, and keywords occurring in at least five publications were retained, yielding 41 keywords in the final network.

The keyword co-occurrence network comprised 41 keywords after thesaurus-based normalization and filtering (Figure 4). PBM was the most frequent and strongly connected term (*n* = 155; total link strength [TLS] = 392), followed by carpal tunnel syndrome (*n* = 59; TLS = 167), pain (*n* = 50; TLS = 147), and nerve regeneration (*n* = 33; TLS = 113).

The network included a compression-related region centered on carpal tunnel syndrome, with associated terms including conservative treatment (*n* = 11; TLS = 34), median nerve (*n* = 6; TLS = 18), and nerve conduction (*n* = 7; TLS = 26). Pain-related terms included pain (*n* = 50; TLS = 147) and neuropathic pain (*n* = 15; TLS = 43).

A separate nerve injury/regeneration-related region included nerve regeneration (*n* = 33; TLS = 113), sciatic nerve (*n* = 20; TLS = 65), peripheral nerve injury (*n* = 16; TLS = 49), expression (*n* = 8; TLS = 31), and Schwann cells (*n* = 5; TLS = 17). Neuropathy-related terms included trigeminal neuralgia (*n* = 17; TLS = 39), neuropathic pain (*n* = 15; TLS = 43), peripheral neuropathy (*n* = 11; TLS = 28), diabetic neuropathy (*n* = 10; TLS = 23), and chemotherapy-induced peripheral neuropathy (*n* = 7; TLS = 19).

Density visualization showed the highest concentration around photobiomodulation, carpal tunnel syndrome, pain, and nerve regeneration (Figure S10). Overlay visualization showed earlier average publication years for irradiation (2013.77), nerve conduction (2015.86), light (2015.83), laser acupuncture (2016.71), and carpal tunnel syndrome (2017.88), whereas more recent terms included chemotherapy-induced peripheral neuropathy (2024.29), pain management (2024.20), paresthesia (2024.00), peripheral nerve injuries (2024.00), trigeminal neuralgia (2022.06), peripheral neuropathy (2021.82), diabetic neuropathy (2021.70), and oxidative stress (2021.50) (Figure S11).

### 3.6. Disease-Specific Keyword Characteristics and Structural Relationships

**Figure 5:**
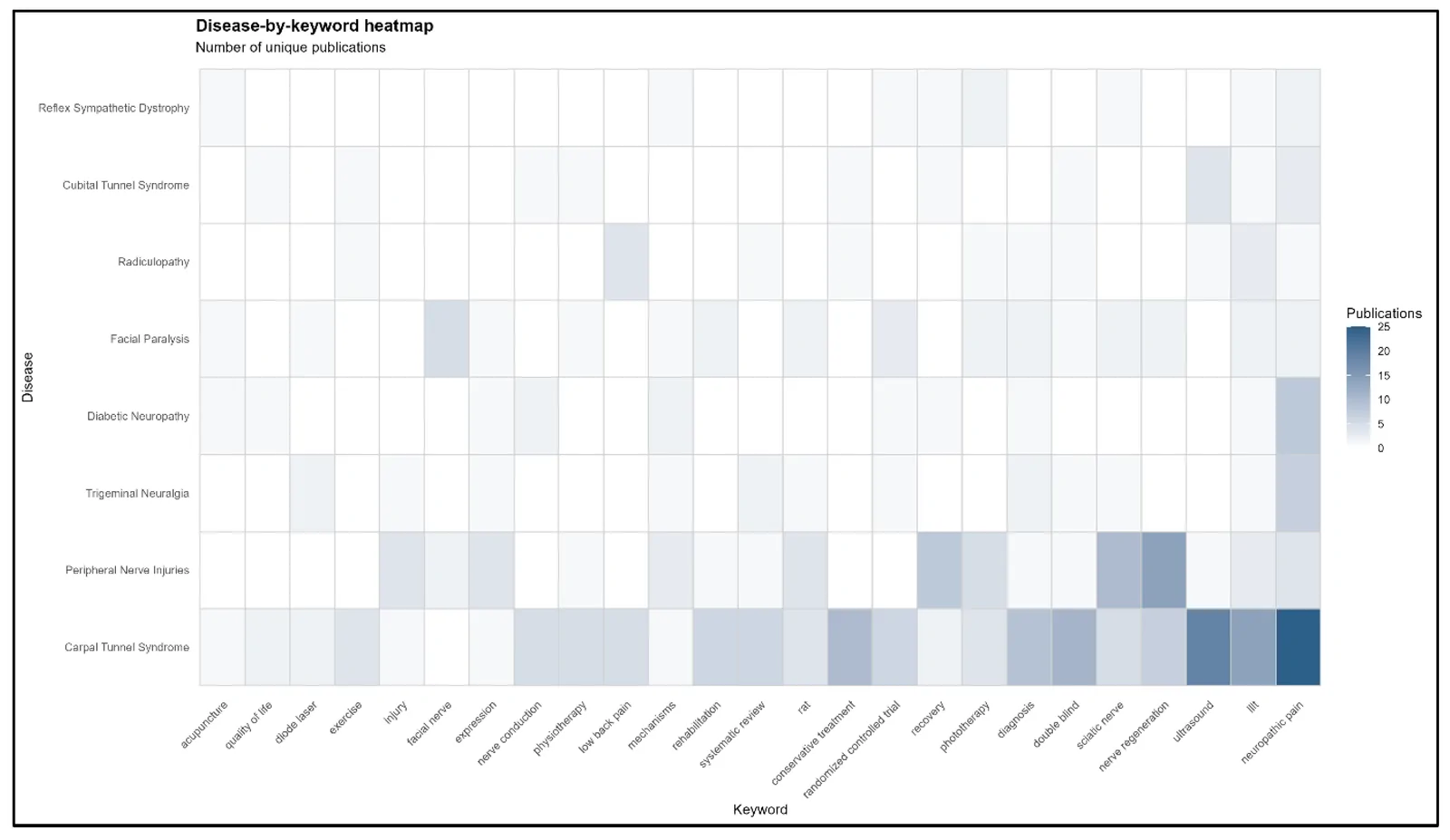
Disease-by-keyword distribution heatmap in PBM research. Each cell represents the number of unique publications containing the corresponding normalized keyword within each disease. Repeated occurrences of the same keyword within an individual publication were counted only once. Color intensity represents absolute publication frequency rather than effect direction or relative disease-specific enrichment.

The distribution of major keywords across individual diseases showed distinct condition-specific co-occurrence profiles (Figure 5 and Figure S12). In carpal tunnel syndrome (CTS), neuropathic pain was identified in 25 of 62 publications, followed by ultrasound (*n* = 19), PBM related terminology (*n* = 17), double-blind design (*n* = 11), conservative treatment (*n* = 10), and diagnosis (*n* = 9). In cubital tunnel syndrome, ultrasound (*n* = 4 of 5) and neuropathic pain (*n* = 3) were the most frequent terms (Figure S12).

In peripheral nerve injuries, nerve regeneration was the most frequent functional keyword (*n* = 14 of 27 publications), followed by recovery (*n* = 8), repair (*n* = 6), phototherapy (*n* = 5), and terms such as expression, Schwann cells, crush injury, and growth factor. For facial paralysis, laser acupuncture was the most frequent functional keyword (*n* = 4 of 27), with randomized controlled trial (*n* = 3), diagnosis, electromyography, nerve conduction study, nerve regeneration, neuropathic pain, oxidative stress, PBM, and phototherapy occurring in two or more publications.

In diabetic neuropathy and trigeminal neuralgia, neuropathic pain occurred in 8 of 18 and 7 of 13 publications, respectively. Diabetic neuropathy was additionally accompanied by mechanisms, nerve conduction, nerve conduction velocity, allodynia, biochemical changes, and biomarkers. Trigeminal neuralgia frequently included diagnosis, diode laser, facial pain, orofacial pain, systematic review, and anticonvulsants. In radiculopathy, low back pain appeared in 4 of 8 publications, followed by PBM (*n* = 3), penetration (*n* = 2), and reliability (*n* = 2).

### 3.7. Comparative Analysis of Functional Research Patterns by Pathophysiological Groups

**Figure 6:**
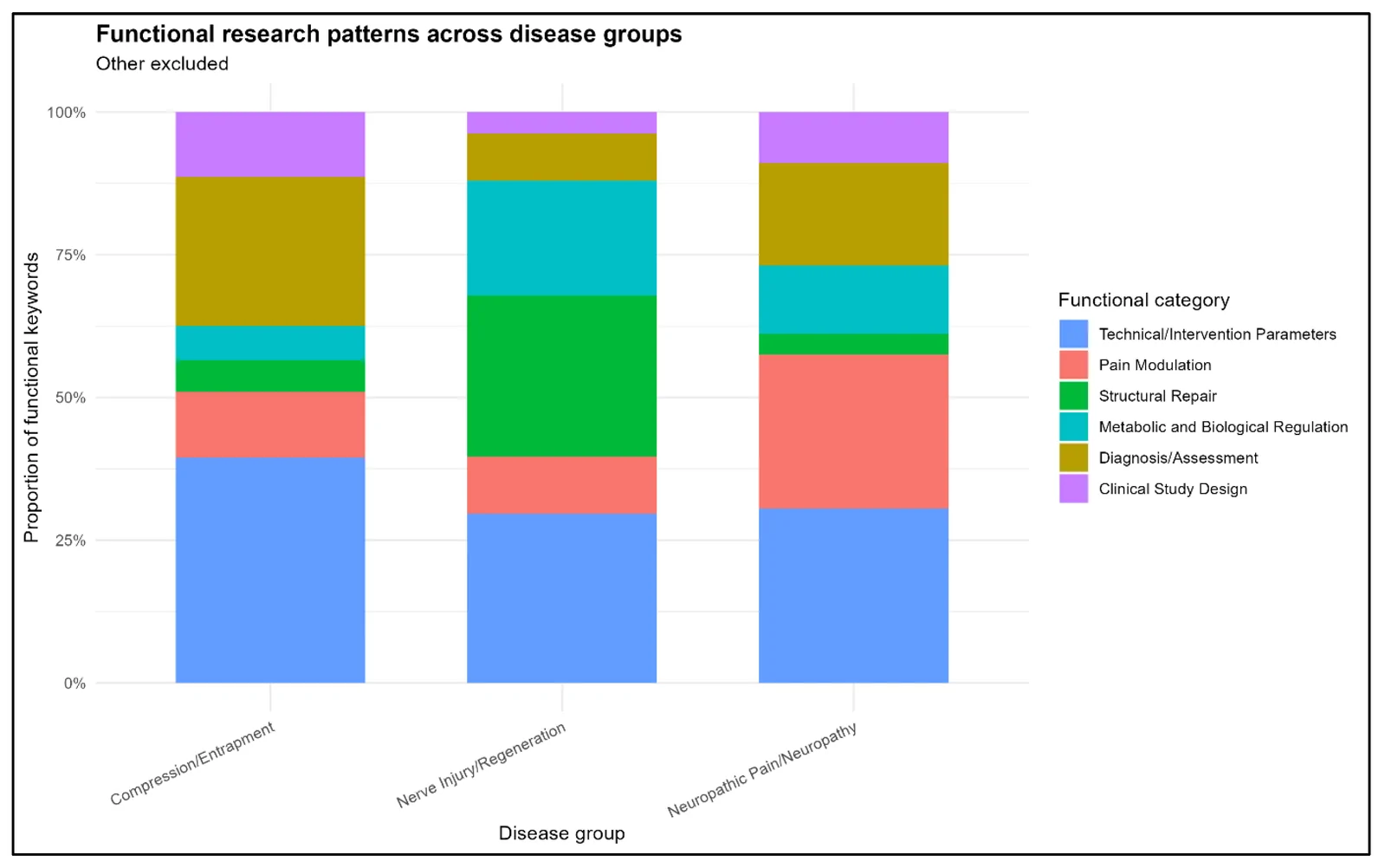
Distribution of functional keyword categories across pathophysiological disease groups. Stacked bars show the proportions of six functional keyword categories within the compression/entrapment, nerve injury/regeneration, and neuropathic pain/neuropathy groups. Proportions were calculated from unique publication–disease–keyword occurrences after excluding keywords classified as “Other” from the denominator.

The distribution of functional keyword categories differed across the three pathophysiological groups (Figure 6). Proportions were calculated from unique publication–disease–keyword occurrences after excluding keywords classified as “Other” from the denominator.

In the compression/entrapment group, technical/intervention parameters accounted for the largest proportion (39.4%; 226/573), followed by diagnosis/assessment (26.2%; 150/573), pain modulation (11.5%; 66/573), and clinical study design (11.3%). Metabolic and biological regulation and structural repair accounted for 5.9% and 5.6%, respectively.

In the nerve injury/regeneration group, technical/intervention parameters (29.6%; 103/348) and structural repair (28.2%; 98/348) were the two largest categories, followed by metabolic and biological regulation (20.1%; 70/348), pain modulation (10.1%), diagnosis/assessment (8.3%), and clinical study design (3.7%).

In the neuropathic pain/neuropathy group, technical/intervention parameters accounted for 30.6% (100/327), followed by pain modulation (26.9%; 88/327), diagnosis/assessment (18.0%; 59/327), metabolic and biological regulation (11.9%; 39/327), clinical study design (8.9%), and structural repair (3.7%; 12/327).

Among the three core therapeutic categories (Figure S13), pain modulation accounted for 50.0% in compression/entrapment, whereas structural repair accounted for 48.3% in nerve injury/regeneration and pain modulation accounted for 63.3% in neuropathic pain/neuropathy.

## 4. Discussion

### 4.1. Summary

This bibliometric study characterizes the research structure and developmental trends of PBM in PNS disorders from 2000 to 2026. While PBM research has expanded steadily since 2010, publication activity remains concentrated around specific conditions, with CTS serving as the most frequently studied clinical condition.

Keyword co-occurrence and functional analyses demonstrated that PBM research is organized around three primary therapeutic axes, pain modulation, neural regeneration/repair, and metabolic/biological regulation, embedded within a broader structure focused on technical parameters and clinical assessment. The relative emphasis among these domains differed across pathophysiological groups: compression neuropathies emphasized technical parameters and assessment, nerve injury research showed greater representation of structural repair and biological regulation, and neuropathy research showed a prominent pain-related component. Temporal trends further indicated diversification from a relatively limited set of earlier clinical conditions toward a broader spectrum of neuropathic disorders, with more recent representation of neuropathic pain, peripheral neuropathies, chemotherapy-induced peripheral neuropathy, and related biological themes.

### 4.2. Emphasis on Treatment Parameters in Compression Neuropathies

In research on compressive and entrapment neuropathies, particularly CTS, technical/intervention parameters and clinical assessment were the two most strongly represented functional categories, whereas structural repair and biological regulation were comparatively less represented. This pattern indicates that PBM research in this domain has focused substantially on treatment conditions, protocol optimization, and clinical evaluation.

Clinical studies on CTS continue to exhibit substantial variability in irradiation parameters such as wavelength, energy density, and treatment frequency, which have been associated with heterogeneity in reported clinical outcomes [9,17]. Although dosage guidelines from the World Association for Laser Therapy (WALT) are available [18], variation in treatment protocols remains a prominent issue in this literature.

The frequent occurrence of ultrasound, conservative treatment, diagnosis, and double-blind design alongside CTS further supports the predominantly clinical and protocol-oriented structure of compression neuropathy research.

### 4.3. Regenerative and Biological Themes in Nerve Injury Research

In contrast to compression neuropathies, nerve injury/regeneration research showed substantial representation of both structural repair and metabolic/biological regulation. Although technical/intervention parameters remained the largest overall category (29.6%), structural repair was similar in magnitude (28.2%) and predominated when the analysis was restricted to the three core therapeutic domains (48.3%). The co-occurrence of nerve regeneration with peripheral nerve injury, sciatic nerve, Schwann cells, expression, and injury-related terms further supports this regenerative research profile. Previous reviews of experimental peripheral nerve injury models have similarly described PBM research in relation to nerve regeneration and functional recovery while emphasizing substantial variability in irradiation parameters [19,20].

Traumatic nerve recovery involves processes including Wallerian degeneration, axonal regrowth, remyelination, and Schwann cell responses [11,12]. Previous experimental studies have linked PBM with mitochondrial activity, Schwann cell responses, axonal regeneration, and myelin formation [11,21], providing biological context for the prominence of regenerative and metabolic terms in this literature. More recent work has also examined PBM in conduit-based peripheral nerve repair, including the potential contribution of vascularization to the regenerative environment following severe peripheral nerve injury [20].

Thus, nerve injury research combines a strong regenerative and biological component with continued attention to PBM intervention parameters.

### 4.4. Pain and Biological Regulation in Neuropathy

Neuropathic pain and neuropathy research showed a comparatively strong representation of pain modulation and metabolic/biological regulation, with structural repair remaining limited. Among the three core therapeutic domains, pain modulation accounted for 63.3%, compared with 28.1% for metabolic/biological regulation and 8.6% for structural repair.

This pattern is consistent with the biological context of disorders such as diabetic neuropathy, which involves chronic hyperglycemia, microvascular damage, oxidative stress, and mitochondrial dysfunction [22,23]. PBM research in this area has examined pathways involving mitochondrial activity, reactive oxygen species, nitric oxide, inflammatory signaling, and analgesic mechanisms [10,24].

The contrast with nerve injury research is notable: whereas structural repair is prominently represented in traumatic nerve injury, neuropathy research is more strongly organized around pain-related and metabolic/biological themes.

### 4.5. Temporal Diversification of PBM Research

Temporal analysis indicates that the evolution of PNS-related PBM research is characterized by thematic diversification rather than a simple transition from clinical to mechanistic research. Earlier research was concentrated around a relatively limited set of conditions, particularly CTS and focal nerve injury, whereas the post-2018 literature encompassed a broader range of compression neuropathies, polyneuropathies, neuropathic pain conditions, and nerve-injury disorders.

Overlay analysis similarly showed relatively recent representation of chemotherapy-induced peripheral neuropathy, peripheral neuropathy, trigeminal neuralgia, diabetic neuropathy, neuropathic pain, and oxidative stress. In contrast, irradiation, nerve conduction, light, laser acupuncture, and CTS showed earlier average publication years. These findings indicate that expansion of the field has occurred across both disease targets and research themes rather than through replacement of earlier topics by a single emerging research direction.

### 4.6. Limitations and Future Perspectives

This study has several limitations. First, analyses based on Author Keywords and Keywords Plus cannot fully capture detailed treatment parameters reported exclusively within full texts. Second, the MeSH- and ICD-11-based classification may omit non-standardized or emerging terminology. Third, reliance on the Web of Science Core Collection may exclude relevant studies indexed elsewhere. Fourth, geographic and institutional concentration may influence the observed publication patterns. Fifth, assigning multifunctional keywords to a single primary functional category necessarily simplifies some conceptual overlap, despite substantial inter-rater agreement (Cohen’s κ = 0.70). Finally, bibliometric co-occurrence reflects literature structure and terminological prominence rather than treatment efficacy or study quality.

Future studies integrating bibliometric patterns with full-text treatment parameters, study design, clinical outcomes, and methodological quality could further clarify how research characteristics differ across PNS disorders.

## 5. Conclusions

This bibliometric study mapped the research landscape of PBM across PNS disorders from 2000 to 2026. While CTS remains the most frequently studied condition, research has diversified across a broader spectrum of neuropathic and nerve-injury disorders. Functional patterns differed across disease groups: compression neuropathies emphasized technical parameters and clinical assessment, nerve injury/regeneration research showed greater representation of structural repair and biological regulation, and neuropathic pain/neuropathy research showed a prominent pain-related component. Temporal analyses further indicated diversification of both disease targets and research themes.

Together, these findings characterize a disease-dependent research structure in the PBM literature and provide a framework for identifying underrepresented areas and future research priorities.

## Data Availability

The data supporting the findings of this study are available from the corresponding author upon reasonable request.

## Supplementary Materials

The following supporting information can be downloaded at: https://www.mdpi.com/article/10.3390/bioengineering13091006/s1, Figure S1: Comprehensive temporal distribution of PBM studies across all peripheral nervous system disorders; Figure S2: Global distribution of publications by country; Figure S3: Geographic distribution of PBM studies by disease and country; Figure S4: Geographic distribution of PBM studies by disease group and country; Figure S5: Distribution of publications across journals; Figure S6: Distribution of PBM studies across journals by disease; Figure S7: Distribution of PBM studies across journals by disease group; Figure S8: Leading institutional contributors to PBM research; Figure S9: Author productivity distribution; Figure S10: Density visualization of keyword co-occurrence network in PBM research; Figure S11: Overlay visualization of keyword co-occurrence network in PBM studies of peripheral nervous system disorders; Figure S12: Top functional keywords within each disease in PBM research; Figure S13: Therapeutic core patterns across disease groups; Table S1: Keyword normalization and filtering scheme for keyword-frequency and functional analyses; Table S2: Thesaurus used for normalization and exclusion in the final VOSviewer keyword co-occurrence analysis.
