# Supplementary material for: Photobiomodulation in Peripheral Nervous System Disorders: A Bibliometric Analysis of Functional Research Patterns
Source: Bioengineering (Basel). 2026 Aug 28;13(9):1006. doi: 10.3390/bioengineering13091006 (PMC13603448; doi:10.3390/bioengineering13091006)

Supplementary materials

Table S1. Keyword normalization and filtering scheme for keyword-frequency and functional analyses

Original keyword	Category	Action / rationale
laser	generic intervention term	excluded because of insufficient specificity
low level laser therapy	synonym	standardized as photobiomodulation
low level light therapy	synonym	standardized as photobiomodulation
low level laser	synonym	standardized as photobiomodulation
photobiomodulation	primary intervention term	excluded from functional-category analysis
photobiomodulation therapy	synonym	standardized as photobiomodulation; excluded from functional-category analysis
pbm	abbreviation	standardized as photobiomodulation; excluded from functional-category analysis
therapy	generic intervention term	excluded because of insufficient specificity
treatment	generic intervention term	excluded because of insufficient specificity
management	generic intervention term	excluded because of insufficient specificity
nerve	generic biological term	excluded because of insufficient specificity
study	generic term	excluded as non-informative
studies	generic term	excluded as non-informative
efficacy	generic outcome term	excluded because of insufficient specificity
patients	generic subject term	excluded as non-informative
patient	generic subject term	excluded as non-informative
disease	generic term	excluded because of insufficient specificity
disorders	generic term	excluded because of insufficient specificity
syndrome	generic term	excluded because of insufficient specificity
article	document descriptor	excluded as non-informative
controlled trial	methodological descriptor	excluded because of insufficient specificity
trial	methodological descriptor	excluded because of insufficient specificity
hand	generic anatomical term	excluded because of insufficient functional specificity
level laser therapy	synonym	standardized as photobiomodulation
laser therapy	synonym	standardized as photobiomodulation
review article	document descriptor	excluded as non-informative
nm	technical unit	excluded as non-informative
irradiation	generic technical descriptor	excluded because of insufficient specificity
light	generic intervention term	excluded because of insufficient specificity
532 nm	specific wavelength term	excluded from functional analysis because of insufficient standalone interpretability
808 nm laser therapy	specific intervention configuration	retained/standardized according to intervention context
blue	generic descriptor	excluded because of insufficient specificity
he ne laser	intervention synonym	standardized as photobiomodulation
llt	abbreviation	standardized as photobiomodulation
lll	abbreviation	standardized as photobiomodulation

Note. Table S1 describes preprocessing applied to keyword-frequency and functional-category analyses. The primary PBM intervention term and generic or non-informative terms were excluded from functional classification to prevent the intervention itself from being interpreted as a functional research category. VOSviewer network-specific normalization and exclusion were performed separately using the thesaurus shown in Table S2.

Table S2. Thesaurus used for normalization and exclusion in the final VOSviewer keyword co-occurrence analysis

Original keyword	Standardized term	Action
Intervention terminology normalization		
low level laser	photobiomodulation	merged
low level laser therapy	photobiomodulation	merged
low-level laser	photobiomodulation	merged
low-level laser therapy	photobiomodulation	merged
low-level light therapy	photobiomodulation	merged
low-power laser	photobiomodulation	merged
laser	photobiomodulation	merged
diode-laser	photobiomodulation	merged
he-ne-laser	photobiomodulation	merged
pbm	photobiomodulation	merged
lllt	photobiomodulation	merged
pbmt	photobiomodulation	merged
light therapy	photobiomodulation	merged
phototherapy	photobiomodulation	merged
laser therapy	photobiomodulation	merged
low power laser	photobiomodulation	merged
low-power laser therapy	photobiomodulation	merged
photobiomodulation therapy	photobiomodulation	merged
low level laser therap	photobiomodulation	merged
level laser therapy	photobiomodulation	merged
Disease normalization		
carpal-tunnel-syndrome	Carpal tunnel syndrome	merged
carpal tunnel syndrome	Carpal tunnel syndrome	merged
trigeminal nerve injuries	Trigeminal neuralgia	merged
orofacial pain	Trigeminal neuralgia	merged
trigeminal neuralgia	Trigeminal neuralgia	merged
peripheral nerve injur	peripheral nerve injury	merged
peripheral nerve injury	peripheral nerve injury	merged
bell palsy	facial paralysis	merged
bell's palsy	facial paralysis	merged
bells palsy	facial paralysis	merged
bells-palsy	facial paralysis	merged
bell-palsy	facial paralysis	merged
Mechanism / concept grouping		
neural regeneration	nerve regeneration	merged
regeneration	nerve regeneration	merged
repair	nerve regeneration	merged
recovery	nerve regeneration	merged
Excluded from global co-occurrence network (noise filtering)		
randomized controlled trial	-	removed
randomized controlled-trial	-	removed
randomized-controlled trial	-	removed
randomized-controlled-trial	-	removed
systematic review	-	removed
meta-analysis	-	removed
placebo	-	removed
double blind	-	removed
double-blind	-	removed
clinical trial	-	removed
therapy	-	removed

management	-	removed
mechanisms	-	removed
pathophysiology	-	removed
diagnosis	-	removed
symptoms	-	removed
outcome	-	removed
evaluation	-	removed
nerve	-	removed
hand	-	removed
muscle	-	removed
ultrasound	-	removed
acupuncture	-	removed
nm	-	removed
wavelength	-	removed
dosage	-	removed
intensity	-	removed
energy density	-	removed
rats	-	removed
stem cells	-	removed
rehabilitation	-	removed
exercise	-	removed

Figure S1. Comprehensive temporal distribution of PBM studies across all peripheral nervous system disorders

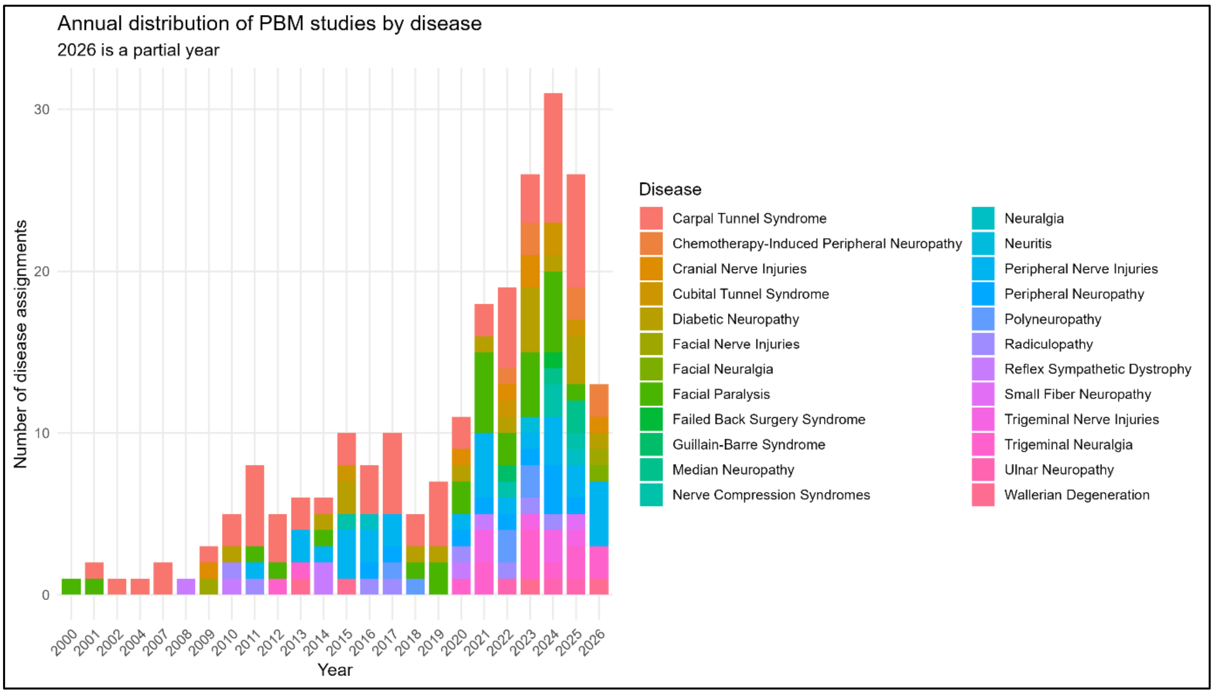

Figure S2. Global distribution of publications by country

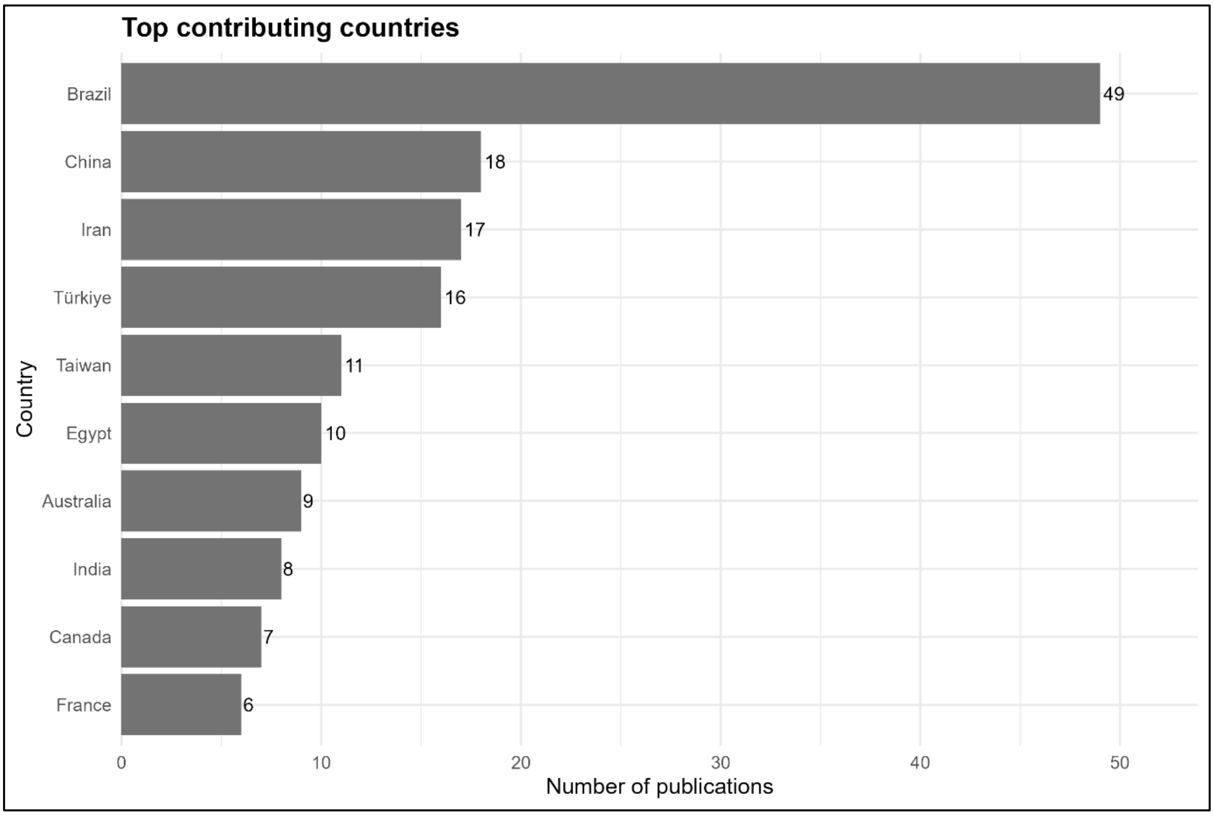

Figure S3. Geographic distribution of PBM studies by disease and country

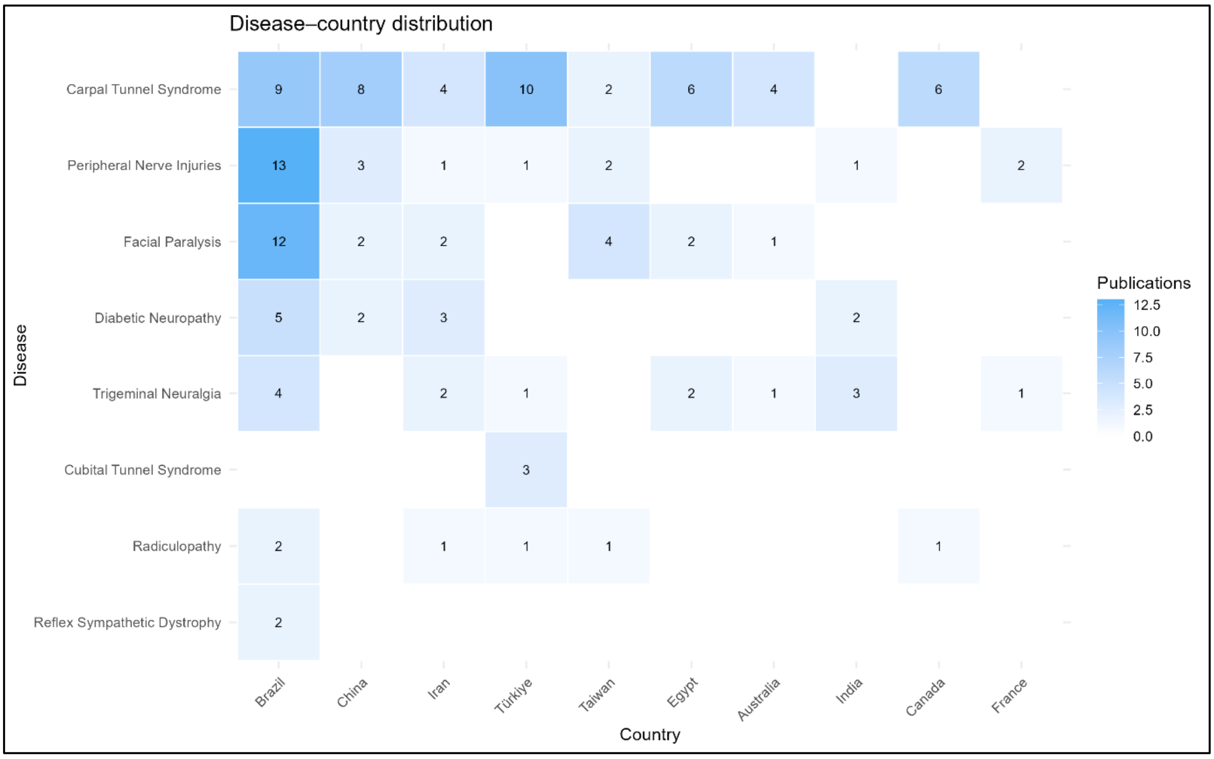

Figure S4. Geographic distribution of PBM studies by disease group and country

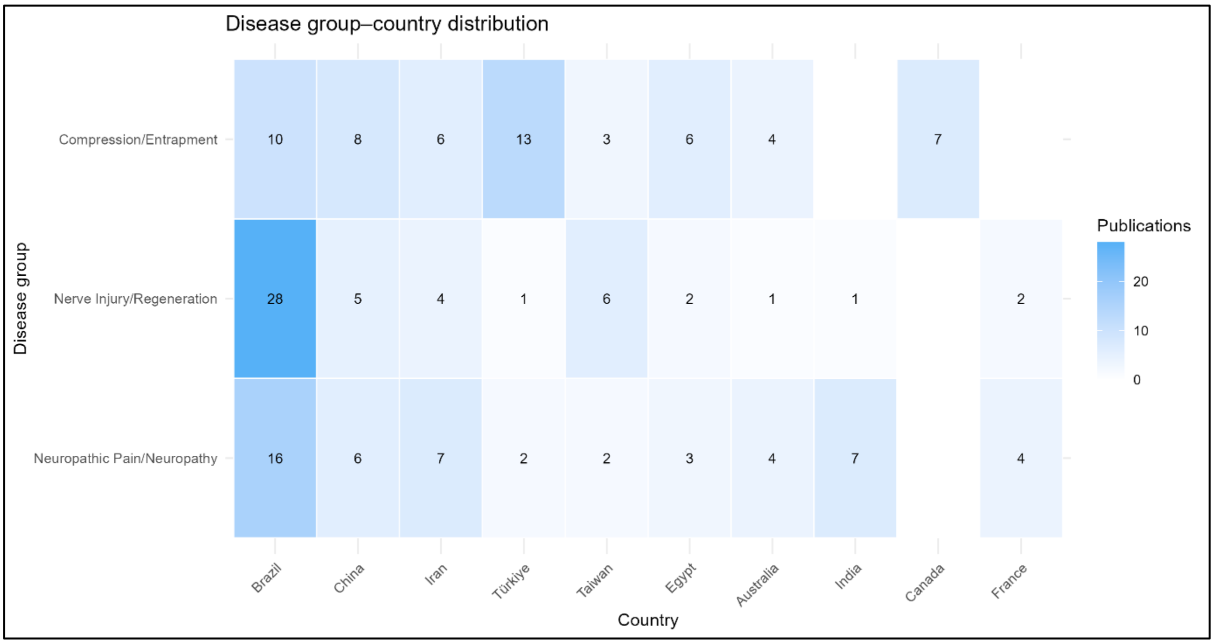

Figure S5. Distribution of publications across journals

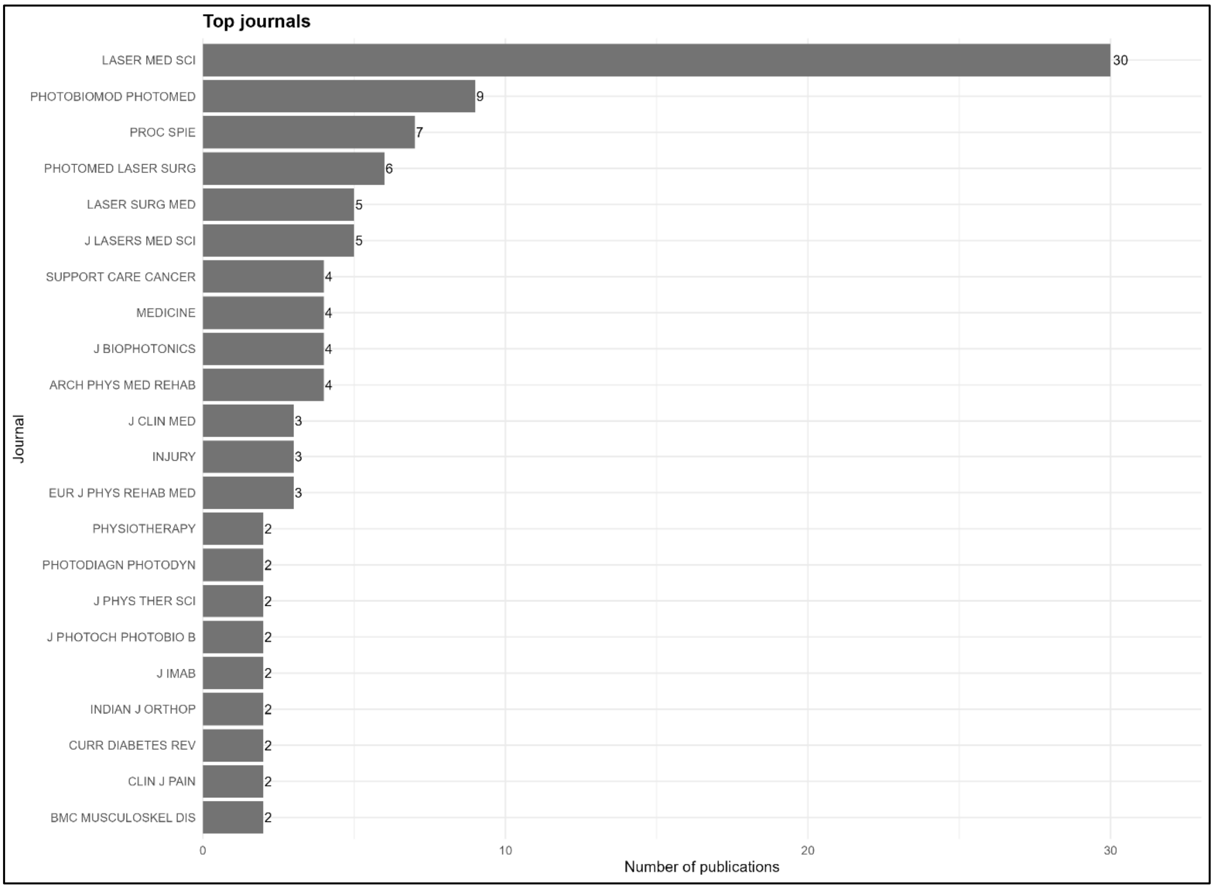

Figure S6. Distribution of PBM studies across journals by disease

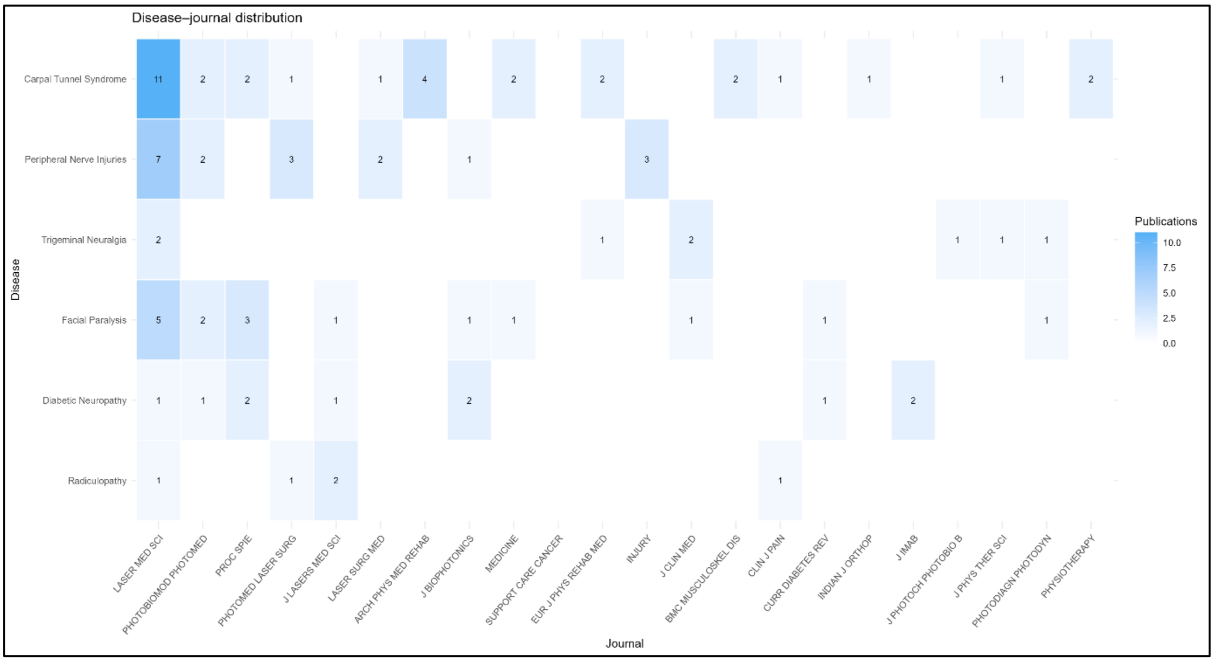

Figure S7. Distribution of PBM studies across journals by disease group

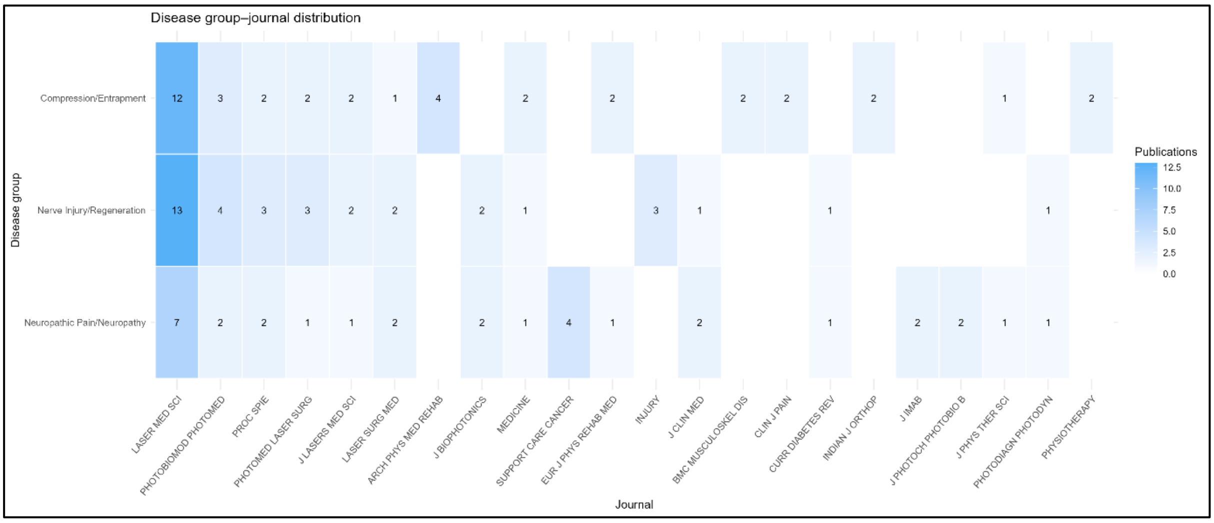

Figure S8. Leading institutional contributors to PBM research

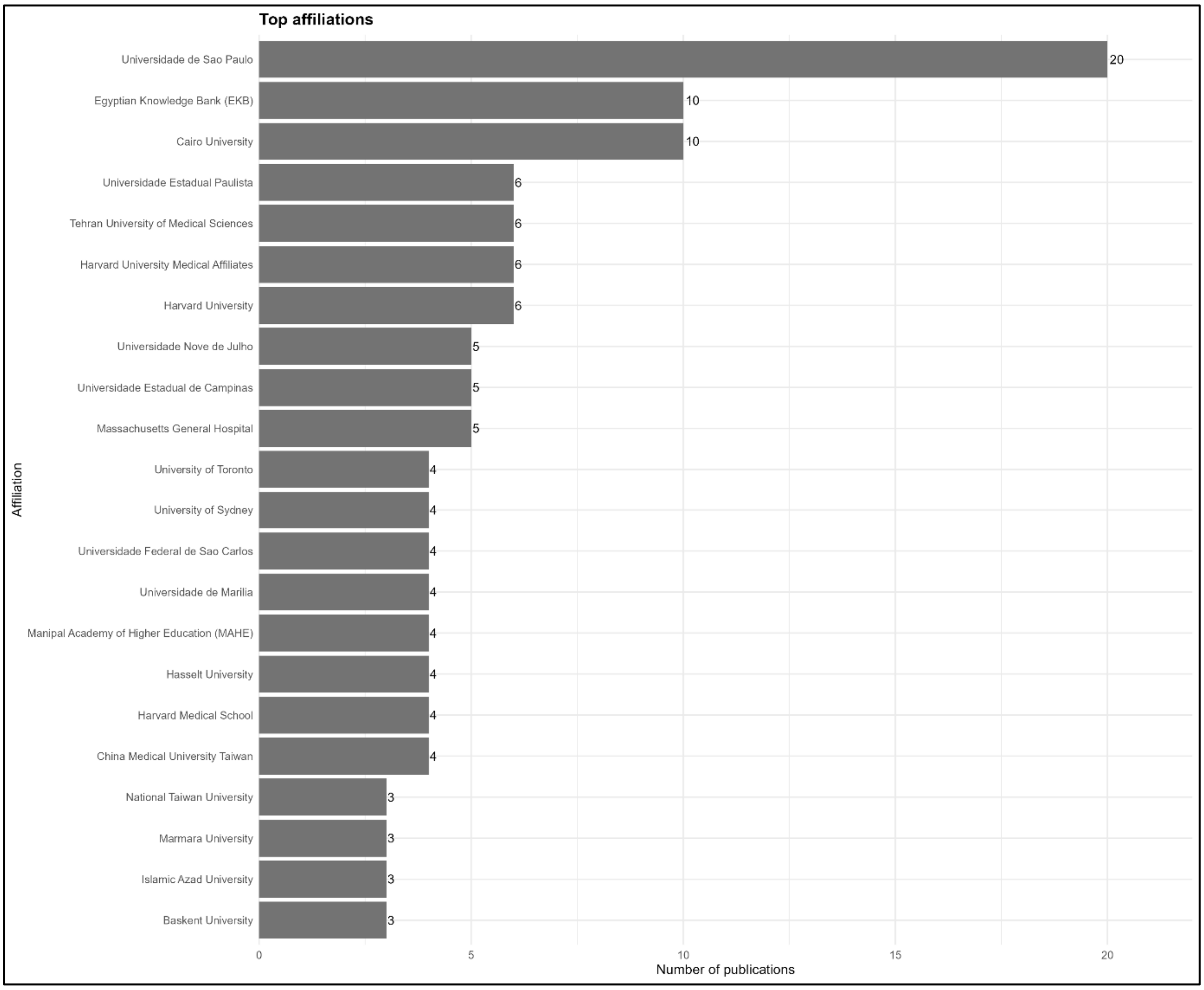

Figure S9. Author productivity distribution

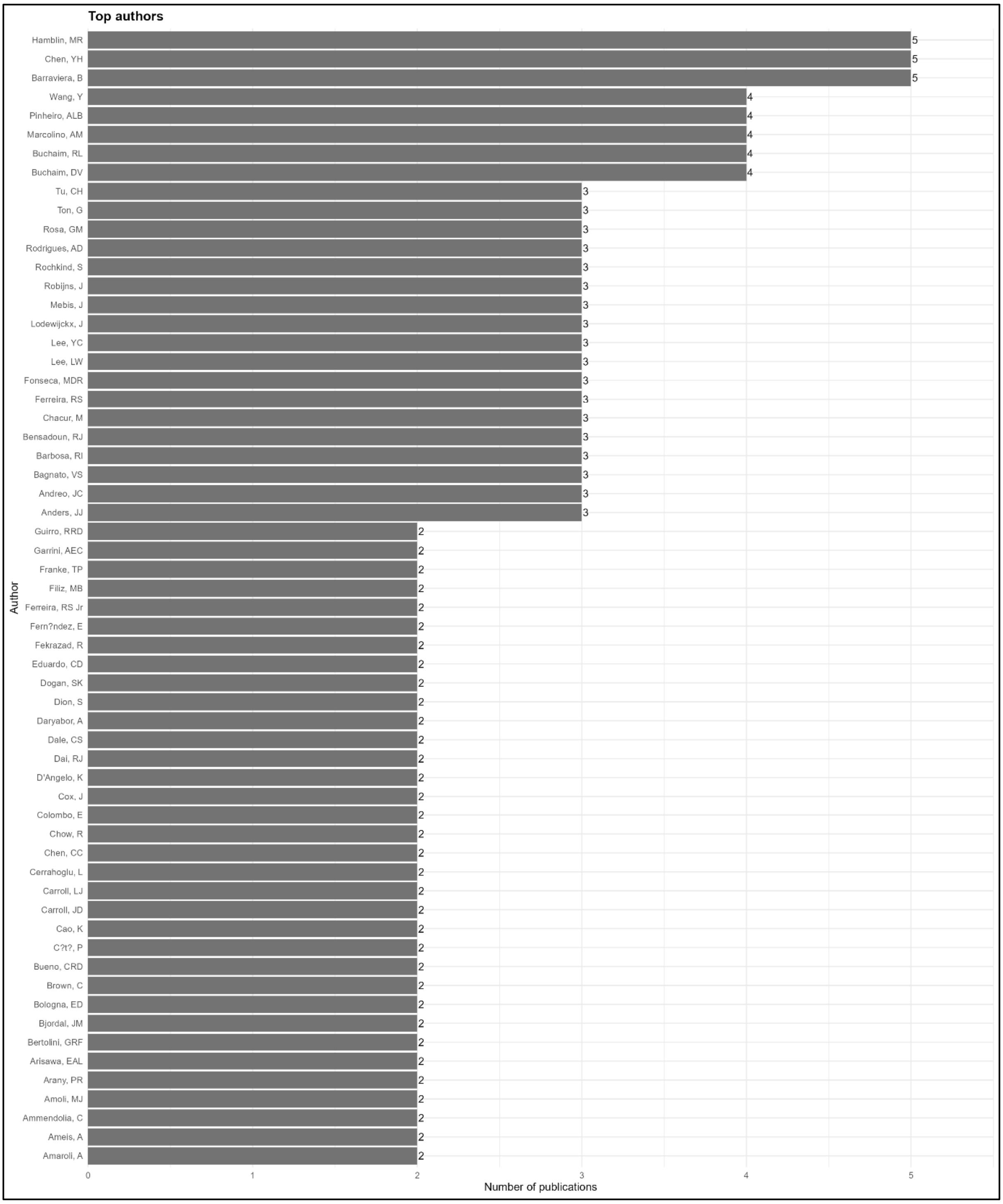

Figure S10. Density visualization of keyword co-occurrence network in PBM research

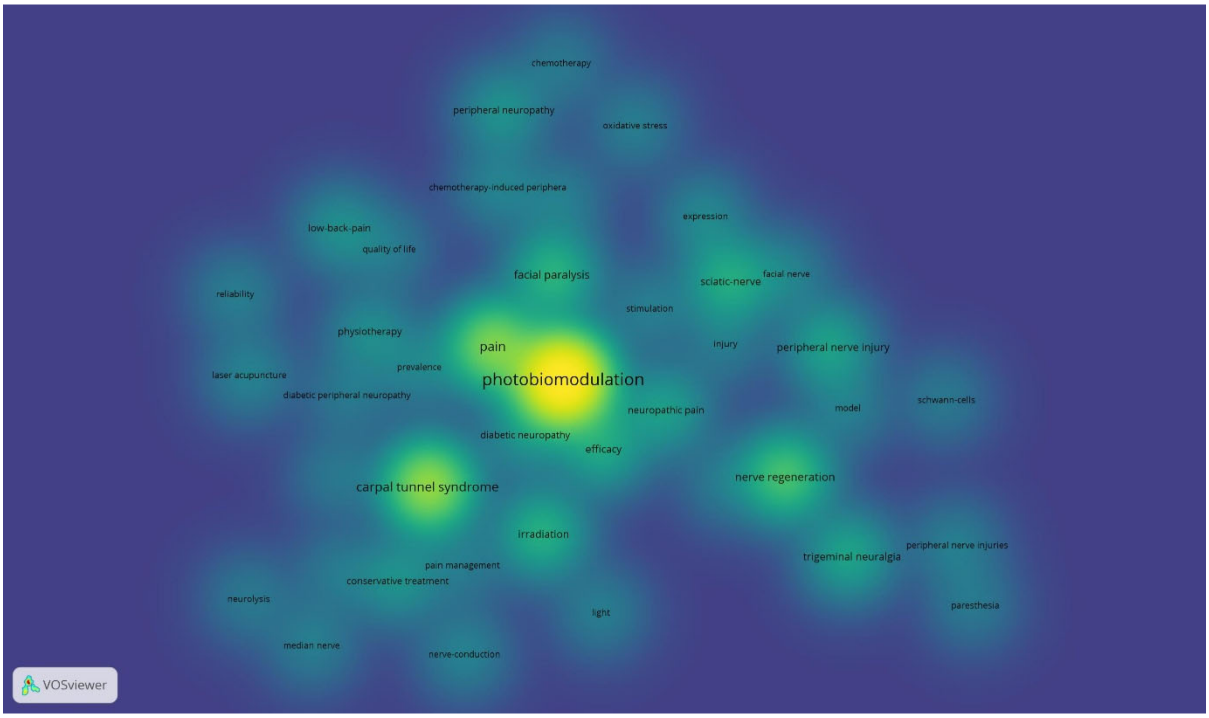

Figure S11. Overlay visualization of keyword co-occurrence network in PBM studies of peripheral nervous system disorders

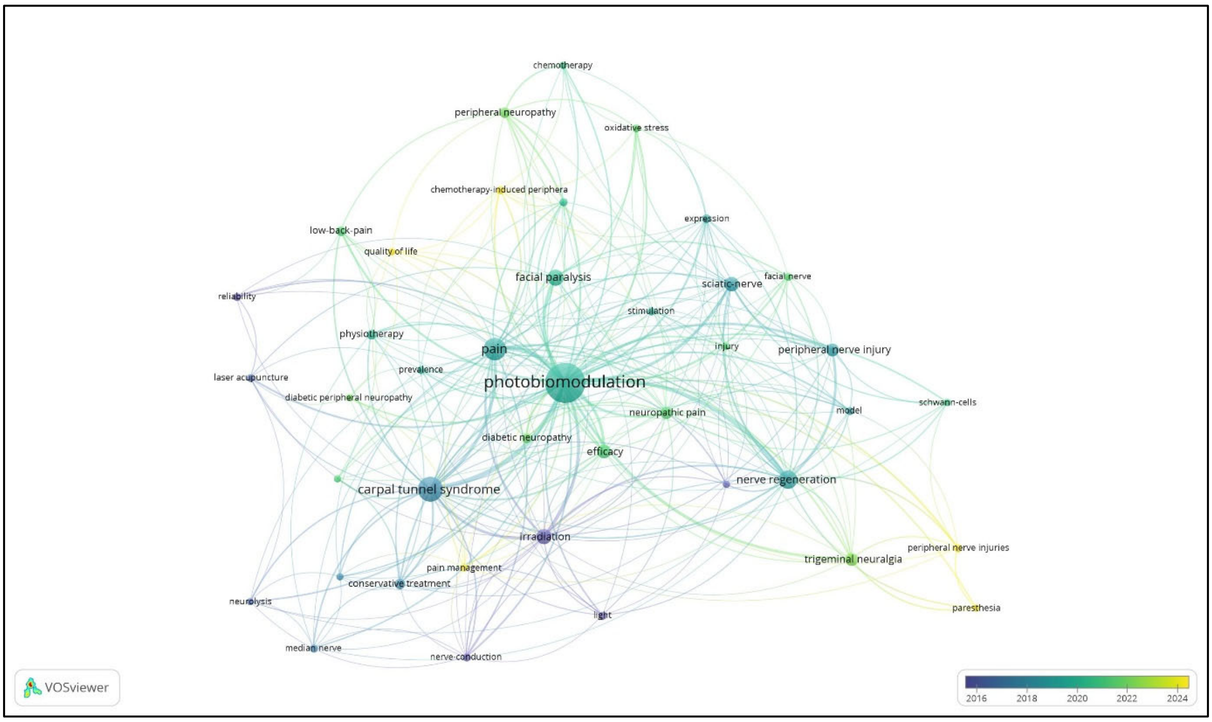

Figure S12. Top functional keywords within each disease in PBM research

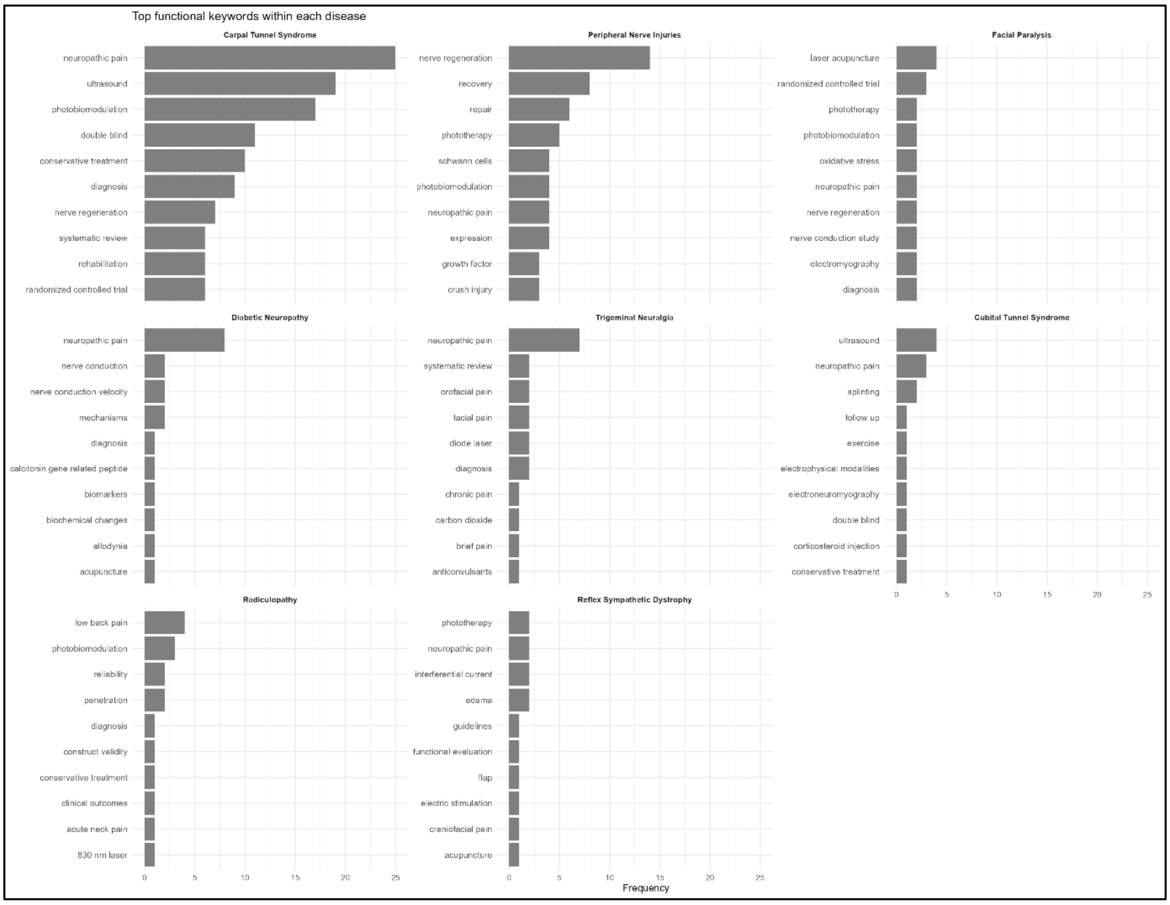

Figure S13. Therapeutic core patterns across disease groups

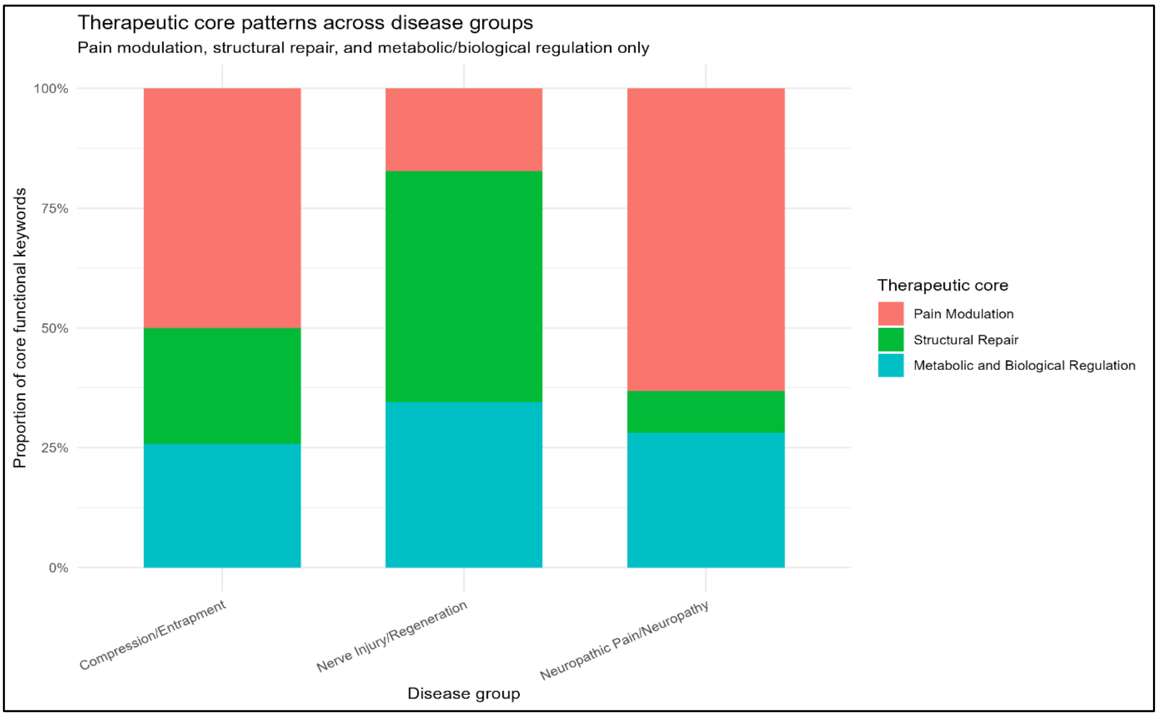

Supplement: Supplementary file 1 [file bioengineering-13-01006-s001.zip › bioengineering-4470378-supplementary.pdf]
